# Letter from the Program Directors

**DOI:** 10.19102/icrm.2023.14039

**Published:** 2023-03-15

**Authors:** Wendy Tzou, William Sauer


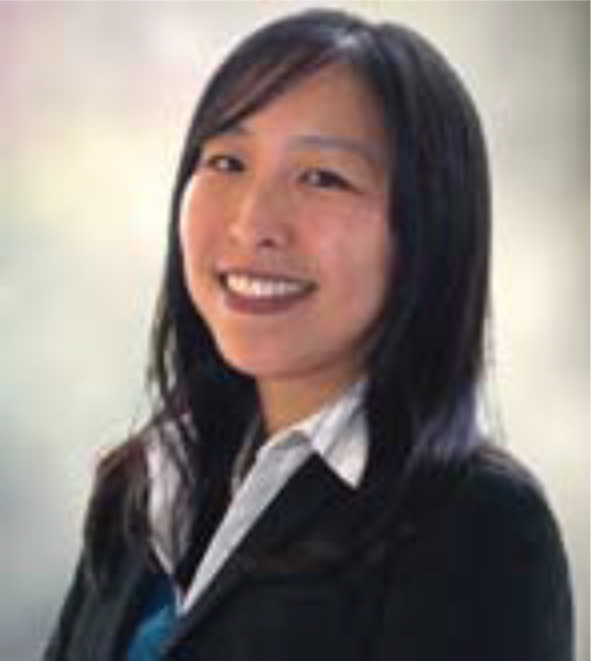



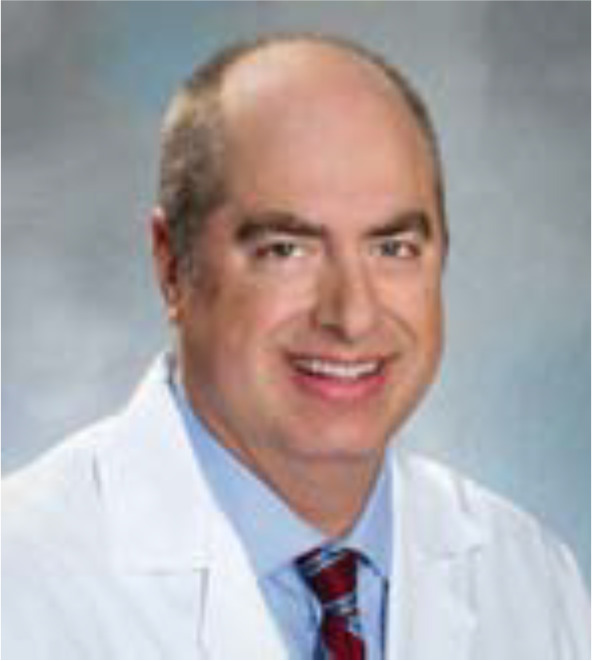


Dear readers,

As the Program Directors for the Electrophysiology Fellows Summit, we are proud to introduce the published case reports of the finalists selected to present their unique cases during the Fellows Competition session at the Electrophysiology Fellows Summit in November 2022.

After a thorough review of the numerous exceptional case entries submitted by fellows and residents, the program committee nominated these finalists to present their work and participate in panel discussions, with the overall winner announced at the sessions’ conclusion following the panel deliberation. (For those who were unable to attend or who wish to view the sessions again, EP Fellows Summit ON-DEMAND at www.epfellowssummit.com provides instant and unlimited access to the full library of educational programming from the Summit.)

Dr. Andrés F. Miranda-Arboleda, a cardiac electrophysiology fellow at Queen’s University in Kingston, Ontario, Canada, described a rare case of syncope associated with swallowing treated successfully with cardioneural ablation. The dramatic presentation included documented episodes of swallowing-associated syncope correlating with several seconds of neurally mediated atrioventricular block. Following mapping and ablation of epicardial ganglionic plexuses in the right and left atria, there was complete elimination of all swallowing-associated symptoms.

Dr. Rohan Trivedi, a cardiac electrophysiology fellow from Geisinger in Pennsylvania, presented a case of a wide complex tachycardia associated with a rare genetic abnormality. A 26-year-old man with a *PRKAG2* mutation, a wide array of electrocardiogram abnormalities, and tachyarrhythmias was successfully managed with an intensive metabolically restrictive diet.

Finally, Dr. Leah John, a research fellow at Brigham and Women’s Hospital in Boston, Massachusetts, presented a case highlighting the use of coronary venous mapping and ablation. She demonstrated effective treatment of ventricular tachycardia using this technique in combination with entrainment maneuvers.

Congratulations to Dr. Miranda-Arboleda on his winning case and to Drs. John and Trivedi as the case competition finalists for their unique and interesting case presentations.

We look forward to your attendance at this year’s EP Fellows Summit, which is scheduled to be held from October 27–29, 2023. As a hybrid conference, attendees will have the choice of attending the Summit virtually or as a traditional in-person event in Charlotte, NC, with the chance to participate in hands-on training sessions and interpersonal engagement

For those who are unable to attend the Summit, virtual attendance and engagement will be made possible from the convenience of your computer or mobile device through the Summit’s innovative live-streaming broadcast platforms. Detailed information will be available at www.epfellowssummit.com.

Sincerely,

Wendy Tzou, md

University of Colorado Anschutz Medical Campus

Aurora, CO, USA

and

William Sauer, md

Brigham and Women’s Hospital

Boston, MA, USA

